# Increasing participation of cancer patients in randomised controlled trials: a systematic review

**DOI:** 10.1186/1745-6215-7-16

**Published:** 2006-05-17

**Authors:** Catriona Mc Daid, Zoé Hodges, Debra Fayter, Lisa Stirk, Alison Eastwood

**Affiliations:** 1 Centre for Reviews and Dissemination, University of York, York, YO10, 5DD, UK; 2 London School of Hygiene and Tropical Medicine, 9 Bedford Square, London, WC1B 3RE, UK

## Abstract

**Background:**

There are many barriers to patient participation in randomised controlled trials of cancer treatments. To increase participation in trials, strategies need to be identified to overcome these barriers. Our aim was to assess the effectiveness of interventions to overcome barriers to patient participation in randomised controlled trials (RCTs) of cancer treatments.

**Methods:**

A systematic review was conducted. Published and unpublished studies in any language were searched for in fifteen electronic databases, including MEDLINE, EMBASE, CINAHL and PsycINFO, from inception to the end of 2004.

Studies of any interventions to improve cancer patient participation in RCTs, which reported the change in recruitment rates, were eligible for inclusion. RCTs and non-randomised controlled trials as well as before and after studies reporting baseline rates specific to the population being investigated were included. Data were extracted by one reviewer into structured summary tables and checked for accuracy by a second reviewer. Each included study was assessed against a checklist for methodological quality by one reviewer and checked by a second reviewer. A narrative synthesis was conducted.

**Results:**

Eight studies were identified that met the inclusion criteria: three RCTs, two non-randomised controlled trials and three observational studies. Six of the studies had an intervention that had some relevance to the UK. There was no robust evidence that any of the interventions investigated led to an increase in cancer patient participation in RCTs, though one good quality RCT found that urologists and nurses were equally effective at recruiting participants to a treatment trial for prostate cancer. Although there was no evidence of an effect in any of the studies, the evidence was not of sufficient quality to be able to conclude that these interventions therefore do not work.

**Conclusion:**

There is not a strong evidence-base for interventions that increase cancer patient participation in randomised trials. Further research is required to evaluate the effectiveness of strategies to increase participation in cancer treatment trials.

## Background

Recruitment of the required number of patients is central to successful completion of a trial. In 2000, the NHS Plan set the target of doubling the total proportion of cancer patients entering clinical trials within three years.[1,2] This target was met by 2004, when almost 11% of people with newly diagnosed cancer participated in trials. [3] However, this remains a small proportion of all cancer patients. Recruitment levels vary between trials. Of 333 public and charity funded cancer randomised controlled trials (RCTs), conducted in the UK between 1971 and 2000, one fifth recruited at least 75% of the planned sample, just over one half did not reach the planned sample size, while one fifth recruited less than 25% of the planned number of patients. [4]

While the research literature fails to identify in a clear, reliable and consistent way the barriers involved in cancer trial participation, themes can be identified.[5] Patient related barriers include preference for a specific treatment, level of knowledge, concerns about randomisation and practical issues such as distance from the clinic and transportation costs. Physician and organisation related barriers include lack of time, poor organisational infrastructure, trials competing for the same patients, identifying eligible patients, lack of awareness of ongoing trials and preference for a particular treatment arm. However, the listing of barriers to participation in cancer trials belies a complex situation. The barriers vary in importance in individual trials and are likely to interact in unique ways for individual trials. These issues have been explored from many different disciplinary perspectives including psychology, sociology, ethics, professional education and health policy.

This systematic review is the second part of a project, funded by the National Cancer Research Network, which considers how participation rates in cancer trials might be improved. The first part of the project was a systematic review of the literature relating to the barriers to participation in cancer trials as perceived by patients and clinicians. [5] In the second part we aimed to investigate the evidence-base for interventions to overcome barriers to trial participation.

We were concerned specifically with strategies to increase the participation of patients in RCTs of cancer treatments. There is likely to be some overlap between strategies that increase participation in cancer treatment and cancer screening and prevention trials. However, many of the issues that an apparently healthy individual needs to weigh up before deciding to participate in a prevention or screening trial would seem to be inherently different from those that need to be considered by an individual with cancer with the option of entering a treatment trial. Similarly, while there is likely to be some overlap in strategies that effectively increase participation in randomised and nonrandomised studies, there are many differences. In particular, there is evidence that being faced with the possibility of being randomised to a treatment arm as opposed to choosing treatment on the basis of patient or clinician preferences raises particular concerns for patients, and indeed sometimes clinicians. [5]

An earlier systematic review of interventions to improve recruitment to research studies considered both mock and real scenarios, as well as patient and non-patient groups. [6] Only studies published before 2002 were included, the authors highlighted the possibility of missed studies and the quality assessment was fairly limited. While studies using a hypothetical scenario may be useful in generating ideas as to what might be effective in a real scenario, any interventions found to be effective in increasing willingness to participate in a hypothetical trial would require subsequent testing in a real scenario. Therefore, the decision was made in the current review to focus exclusively on interventions directed at real trials. We were specifically interested in actual trial participation. Patient knowledge and understanding[7] or the quality of clinician communication with patients about RCTs[8] are important outcomes in their own right. However, improvement in these outcomes does not necessarily translate into increased patient participation in cancer trials.[9]

We carried out a systematic review of the available evidence on the effectiveness of any interventions to increase cancer patient participation in randomised controlled trials.

## Methods

### Search strategy

We searched fifteen databases for published and unpublished studies, with no language restrictions: MEDLINE, EMBASE, CINAHL, PsycINFO, Cochrane Database of Systematic Reviews, Cochrane Database of Methodology Reviews, Database of Abstracts of Reviews of Effects, Health Technology Assessment database, American Society of Clinical Oncology Website, Health Management Information Consortium, System for Information and Grey literature in Europe, ISI Science Citation Index, SI Social Science Citation Index, Sociological Abstracts, Applied Social Sciences Index and Abstracts. The search strategy combined groups of search terms representing cancer trials and patient participation. (See Additional file 1 for full details of the MEDLINE search strategy which was amended as necessary for the other databases searched.) We also searched the reference lists of all retrieved articles.

### Selection of eligible studies

Two reviewers independently assessed titles and abstracts and full papers, where these were obtained. We included studies of any interventions to improve cancer patient participation in RCTs, which reported change in participation rates. The primary outcome of interest was patient participation. Therefore, for example, interventions aimed at increasing physician participation in trials, or interventions targeted at organisational change were eligible for inclusion provided the impact on patient participation rates was also assessed. The definition of participation varied between studies and the definition used by individual studies was accepted. We included controlled trials and also before and after studies, provided baseline participation rates were reported specific to the population being investigated. We contacted authors for clarification when it was unclear whether the intervention was directed at randomised or nonrandomised clinical trials.

One reviewer extracted data from included studies into structured summary tables and assessed the methodological quality of the studies. A second reviewer checked the data and quality assessment with disagreements resolved by discussion and consensus. We developed separate quality checklists to assess RCTs, for each relevant study design, based on CRD Report No.4.[10] We assessed whether measures had been taken by the study authors to avoid or minimise selection bias, attrition bias, performance bias and whether the study design protected against contamination between the intervention and the comparison. Studies were also assessed as to whether the nature of the intervention was clear and whether the target of the intervention was clearly defined. It was not appropriate to undertake a statistical synthesis as the included studies were very diverse; therefore we conducted a narrative synthesis.

## Results

### Overview

**Figure 1:**
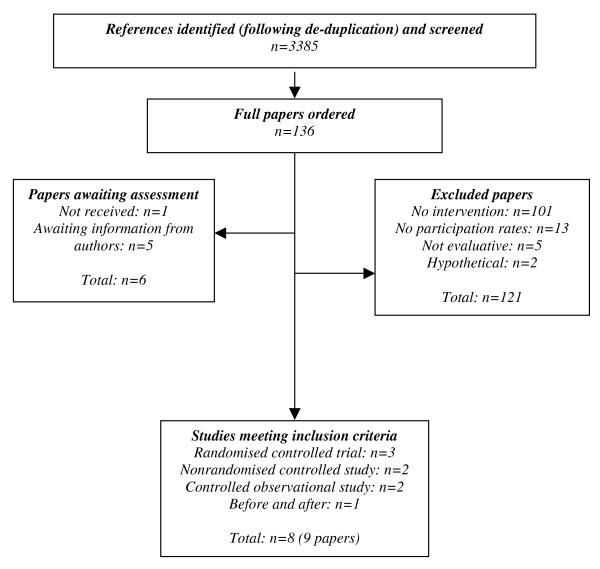
Study selection process.

We screened 3385 references and assessed 136 full papers. Eight studies met our inclusion criteria: three RCTs; two nonrandomised controlled prospective studies; two observational prospective studies with a comparison group and one before and after study (see Figure 1). The majority of studies were excluded because there was no intervention targeted at improving patient participation in cancer trials. Three studies were excluded because data on participation in randomised and non randomised trials were reported together and, following contact with the authors, no separate data were available for patient participation in RCTs. [11-13] It was not possible to assess six papers: one had not been received and for five papers it was unclear whether the intervention was directed at an RCT. [14-18]

### Study characteristics

Table 1 summarises the main characteristics of the eight included studies. We found three studies that had been conducted in the UK or Europe, [19-21] one of which was an RCT.[19] Two of the UK studies were concerned with participation in the same cancer treatment trial.[19,20] The remaining five studies were conducted in the US [22-25] and Australia.[26] The majority of studies were concerned with some aspect of the consent process. In four of the seven studies of adults, the majority of participants were women. Four studies included predominantly white participants[22,23,25,26] and this information was not reported in the remaining studies. The majority of studies included individuals with different cancers. Only two studies provided details of disease stage or severity and only for subgroups of patients.[22,23] The included studies varied in quality. A summary of individual study quality is provided in Table 1 and the full quality appraisal for individual studies is available in the full report.[27] Care needs to be taken when comparing rates of trial participation between studies due to between study variation in how participation was defined. In four studies, trial participation was defined as the number of patients accrued or enrolled but it was not clear whether this referred to the proportion who agreed to randomisation or the proportion who actually accepted their allocation. [22-25] One study that used both measures illustrated that there can be a considerable difference between these figures, with actual accrual being lower than consent to randomisation.[20] A further study based participation rates on questionnaires completed by patients, following their meeting with the doctor, stating whether they wanted to participate. This may have overestimated the number who actually started the trial.[21]

**Table 1:** Characteristics of included studies

**Study details**	**Study design**	**Target of intervention (who received the intervention; and the number of trials across which it was assessed)**	**Patient Participants**	**Experimental Intervention/and comparator**	**Summary of threats to validity**
Angiolillo et al. (2004)[24] United States	Controlled observational study	Parents of children with cancer Four Children Cancer Group Trials	I: n = 36; C: n = 104 Parents of children with acute leukaemia Age of children I: Mean 4.9 yrs (SD 2.5); C: 7.8 yrs (SD 5.1) Ethnicity not stated	**Intervention: **A two-stage process was used for one trial. 1. Written parental consent was sought for the induction phase of the trial during which all patients received the same induction chemotherapy. Written consent (4 weeks later) was then obtained for randomisation to one of four therapeutic regimens. **Comparator: **Parents of children in the other three trials did not receive the staged approach. No further details provided.	A high possibility of selection bias due to lack of randomisation and no reported process for selecting individual participants. The intervention was not implemented in a standardised way. Due to poor reporting the risk of contamination was unclear. There was a particular risk of performance bias.
Coyne et al. (2003)[22] United States	Cluster randomised controlled trial	Adult cancer patients Three trials (C9741; E1594; E2197)	I: n = 78; C: n = 129 Breast (85%) and lung cancer patients I: 92.3% female; C 90.7% female I: Mean 53 yrs; C: mean 53 yrs I: 94% white; C: 92% white	**Intervention: **Easy to read version of the original written consent document (different for each of the three trials). Changes included text style, page layout, font size and vocabulary. Content was not altered. Readability was seventh to eighth grade level and length was 16 pages. **Comparator: **Original consent document (different for each of the three trials). E1594: 4 pages long and fourteenth grade reading level. C9741 and E2197: 7–8 pages long and twelfth to thirteenth grade reading level.	This was a randomised study. The unit of randomisation was at the institutional level and this was maintained for the statistical analysis. However, due to poor reporting it is unclear whether the study was properly designed to protect against selection bias. The design appeared to protect against contamination as only one consent statement was used at an individual centre.
Donovan et al. (2003)[19] United Kingdom	Randomised controlled trial	Adult cancer patients Single trial	I: n = 75; C: n = 75 Prostate cancer patients 100% male Age not stated Ethnicity not stated	**Intervention: **Nurse conducted information appointment with the patient to recruit to the trial. **Comparator: **Urologist conducted information appointment with the patient to recruit to the trial.	A good quality RCT: appropriate randomisation, concealed allocation, at least 80% of patients considered at follow-up and ITT analysis conducted. It is possible that contamination between the two groups and performance bias may have influenced the findings.
Donovan et al. (2002)[20] United Kingdom	Before and after	Healthcare professionals Single trial	Baseline: n = 30; I1: n = 45; I2 n = 67; I3: n = 83; I4: n = 155 Prostate cancer patients 100% male Age not stated Ethnicity not stated	**Intervention: **Three successive documents§ regarding how best to recruit patients to the trial were circulated to recruiters followed by a training programme. Consent to randomisation was measured at baseline and following circulation of each document.	This is an uncontrolled study therefore there is a risk of factors other than the intervention influencing patient participation.
Fleissig et al. (2001)[21] United Kingdom	Nonrandomised controlled study	Healthcare professionals and adult cancer patients Forty trials	I: n = 135; C: n = 130 10 different cancers I: 72% female; C: 72% female Age range 19–65 yrs I: 58% 45–64 yrs; C: 50% 45–64 yrs Ethnicity not stated	**Intervention: **Patients completed the Patient Preferences for Information Questionnaire, Patient Attitudes to Trials Questionnaire and Spielberger State Trait Anxiety Inventory prior to consultation with their doctor. Doctors were then provided with each patient's completed questionnaires (only the first 2 questionnaires) prior to their consultation during which consent was sought for a specific trial. **Comparator: **Patients completed the same questionnaires prior to consultation with their doctor. Doctors were not provided with this information prior to their consultation with individual patients during which consent was sought for a specific trial.	A high possibility of selection bias: only the order in which doctors conducted intervention and control group consultations were randomised (in blocks of 5 patients). The process by which patients were selected for inclusion was not reported. There was a high possibility of contamination as the same doctors were involved administering the experimental intervention and the comparison. The intervention was not implemented in a standardised way. The process of completing the questionnaires may have influenced patient decision-making in both groups.
Gross et al. (2004)[25] United States	Controlled observational study	System level Global target (National Cancer Institute phase II and III Clinical Trials Cooperative Group trials)	I: n = 4569; C: n = 20,443 (2,440 were in phase II trials) Breast, colon, lung and prostate cancer patients Sex not stated Age not stated 89% white	**Intervention: **Four states (Illinois, Louisiana, Virginia, New Jersey) that enacted legislation or developed a co-operative agreement with health insurers in 1999 to cover clinical trial patient care costs (coverage states). **Comparator: **35 states that had not enacted any policies to cover clinical trial patient care costs by the end of 2001 (non-coverage states)	The baseline enrolment rate was statistically significantly higher in intervention group than the comparator group introducing the risk of regression to the mean. Lack of enforcement in the intervention group and behaviour of physicians in the comparator states to compensate for lack of coverage could have had an influence.
Paskett et al. (2002)[23] United States	Nonrandomised controlled study	Adult cancer patients, healthcare professionals All trials available to patients in a given geographical area.	Total number of participants not stated Breast and colorectal cancer patients Majority female Age not stated for I and C (mean age, which was reported by time period of recruitment and cancer type ranged from 62 to 75 yrs) 75% white	**Intervention: **There were four elements: 1) a rapid tumour reporting system, 2) a nurse facilitator responsible for alerting physicians about appropriate clinical trials for their patients, 3) a quarterly newsletter about cancer treatment and clinical trials targeted at physicians and 4) a health educator who provided community-based education about screening and treatment and trained lay health educators. Implemented in five rural counties in North Carolina. **Comparator: **No intervention in five rural counties in South Carolina.	The risk of selection bias is unclear. Data on patient trial participation were obtained from medical records; however it was unclear how specific cancer patients within regions were selected or whether all cases were detected. The study was susceptible to contamination: improving participation of patients in all rural areas was a major focus of the Community Clinical Oncology Program (CCOP) and both geographical areas had active CCOP physicians.
Simes et al. (1986)[26] Australia	Randomised controlled trial	Adult cancer patients and healthcare professionals Thirteen trials at a single oncology unit	I: n = 28; C: n = 29 8 different cancers I: 82% female; C: 62% female I: mean 56 yrs (31–63 yrs); C mean 55 yrs (40–74 yrs) I: 96% white; C: 100% white	**Intervention: **Uniform policy of total disclosure of all information relevant to the trial to the patient. There was an opportunity to ask further questions. Information was provided verbally and in a written consent form. **Comparator: **Information about the aims, anticipated results and potential toxicities of treatment were provided with details of treatment provided at the discretion of the consultant. There was an opportunity for the patient to ask questions. Verbal consent was obtained.	This was a randomised study though it was not possible to assess from the information reported whether the method of assignment was truly random and whether it was concealed. There was a high possibility of contamination as the same doctors were involved in delivering the experimental. Attempts were made to establish whether the intervention and comparison were standardised across patients though it was not possible to establish whether the method used was sufficiently rigorous.

I: experimental intervention, C: comparator

§Document 1 asked recruiters to present the three treatment options in a particular order and provide equivalent detail on advantages and disadvantages. They were asked to avoid the terms trials and 'watchful waiting' to describe monitoring and place emphasis on patients being eligible for all treatments; document 2 re-emphasised monitoring as an active process, eliciting and challenging patients' views if at odds with the evidence and re-emphasised there was no compulsion to accept treatment allocation; document 3 provided good and not so good examples of how to present information about treatments in an equal way.

We synthesised two studies separately from the others as the interventions could not be implemented in the UK; these are only briefly summarised here. One was an RCT conducted almost 20 years ago in Australia which investigated a uniform policy of full disclosure of all relevant information when seeking patient consent to trial participation compared with disclosure of information at the discretion of the consultant[26] A written consent document was completed for the former condition whereas only verbal consent was obtained for the latter. In the UK setting, clinical trials regulations require full disclosure of information with written consent.[28] The second study investigated the effect of legislation requiring health insurers to cover clinical trial patient care costs on trial participation rates in the US.[25] Although the funding of trials is an important issue this study is only relevant in settings where health insurance is widespread.

### Efficacy of interventions

Across the remaining six studies there was no evidence that any of the experimental interventions evaluated led to an increase in cancer patient participation in RCTs compared with the comparison intervention (see Table 2). A good quality RCT, conducted in a UK setting, did find that nurses and urologists were equally effective in recruiting men with prostate cancer to a treatment trial with a two and three-arm comparison though recruitment levels varied between the three centres (94%, 61% and 45%).[19] Based on a cost minimisation analysis, recruitment by nurses was more cost-effective. This finding was unchanged in six out of seven sensitivity analyses exploring different resource scenarios, though the size of the cost difference did change. An uncontrolled qualitative study involving patients and recruitment staff was also undertaken in relation to the same trial. This study found increased participation rates following amendments to the nature and emphasis of information provided to potential trial participants.[20] However, given that this was not a controlled study, the influence of other factors on the recruitment rates cannot be excluded.

**Table 2:** Participation rates

**Study details**	**Intervention**	**Comparator**
Angiolillo et al. (2004)[24]	77%	88%*
Coyne et al. (2003)[22]	75%	68%*
Donovan et al. (2003)[19]	67%	71%*
Donovan et al. (2002)[20]	Baseline 30–40% Intervetion 1 51% Intervention 2 58% Intervention 3 61% Intervention 4 70%	No comparator
Fleissig et al. (2001)[21]	81%	74%*
Paskett et al. (2002)[23]	Breast 1991 15% (n = 24); 1996 6% (n = 14) Colorectal 1991** 4% 1996 5%	Breast 1991 6% (n = 6); 1996 50% (n = 16) Colorectal 1991** 5% 1996 0%

*only those studies with an asterisk assessed statistical significance, all of these were non significant.

** number of patients not available

The following interventions were not associated with an increase in trial participation: a two-stage process for seeking parental consent for their child's participation in a leukaemia trial compared to the standard approach;[24] a written consent document designed to be easy to read compared with the standard consent form; [22] providing doctors with information on patients' individual information needs and attitudes to trials prior to seeking consent compared with the doctor not having this information;[21] and a multi-component, system level intervention compared with no intervention.[23] However, the evidence is not sufficient to conclude that the interventions investigated are ineffective.

In most of the studies participation levels were high in both the intervention and the control group. Apart from one study with low participation levels, [23] participation rates in the control groups ranged from 68% to 88%. This raises the question of whether there was a Hawthorne effect i.e. that the experience of participation in a study per se led to an increase in participation in the cancer trial. This could have been sufficient to mask an effect of the experimental intervention, especially given the fairly small sample sizes in these studies. Alternatively, the particular cancer trials to which patients were being recruited in these studies may have been trials which were easy to recruit to and that would have had high recruitment levels anyway. This may have lead to a ceiling effect in individual trials. None of the interventions appeared to be in response to recruitment problems being encountered either with the specific trials where individual trials were being targeted or with the health professionals conducting the recruitment where a large number of different trials were involved. For two studies related to a trial for treatment of prostate cancer the trial was described as controversial and difficulties in recruitment were anticipated. [19,20]

There is the possibility that the specific interventions investigated do not work in the particular contexts in which they were used. They may prove effective with a different patient group or in relation to a different trial. For example, if the effect on participation levels of an 'easy to read' informed consent form, as used in the study conducted by Coyne et al., [22] had been investigated with patients with a lower level of literacy than the women in the study, it may have been found to be effective.

Most of the studies had methodological weaknesses. Apart from one good quality RCT with appropriate randomisation and concealment of allocation, [19] the remaining studies were vulnerable to selection bias. Across the studies there seemed to be a risk of underestimating the effect of the interventions due to the possibility of contamination between the experimental and comparison intervention. Contamination refers to the situation where the control or comparison group receives part of the experimental intervention leading to unplanned similarities between the two conditions; for example, where the same clinicians are responsible for delivering the experimental intervention and comparison, knowledge of the experimental intervention may influence how the comparator is delivered. This can lead to a dilution of any effect of the experimental intervention. Apart from one included study that minimised the risk through study design, [22] there was a risk of contamination across all studies.

## Discussion

Overall there is not a strong evidence-base for interventions that increase patient participation in cancer treatment trials. Despite the large volume of research that exists on barriers to participation in cancer trials[5], we found only a small body of research on interventions to overcome these barriers. And despite the plethora of potential barriers to participation that have been identified, only a small number of barriers were addressed. Most of the interventions were concerned with the consent process, though the specific aspects addressed varied, ranging from a fairly simple intervention to make a consent form more readable to more complex approaches tailoring the information provided to potential participants. In addition, they were mainly pragmatic interventions; this mirrors the research on barriers to participation in cancer trials which is generally not theoretically driven. [5]

There was no evidence that any of the interventions investigated led to an increase in cancer patient participation in clinical trials. However, the evidence was not of sufficient quality to be able to conclude that these interventions therefore are not effective. Overall the studies had a range of methodological weaknesses and in most of the studies there was a risk of the effect of the intervention being underestimated. The barriers to recruitment may be numerous, complex and probably interact in a unique way in relation to individual trials. In contrast, most studies investigated interventions targeted at one aspect of recruitment in isolation. This is not surprising as it is probably the most straightforward way to evaluate an intervention. However, if the intervention did not target the key barrier to participation in a particular trial, it may not show any evidence of effectiveness in that particular situation. Indeed, some cancer trials experience rapid and successful recruitment, which may relate, for example, to the particular treatment being investigated.[29]

The findings of this systematic review are similar to previous systematic reviews with an overlapping scope. In one review of interventions to increase participation in mock and real trials, in healthy individuals and all patient groups, over 75% of the included studies found no evidence of an effect on participation.[6] In a review of interventions to improve research participants' understanding during the informed consent process a similar proportion of studies found no evidence of an effect on accrual to real or mock trials.[7] The quality assessment in both reviews was fairly limited and possible reasons for the lack of effect in so many of the studies were not explored.

### Strengths and weaknesses of the review

We searched for evidence from a wide range of sources on any interventions targeted at improving patient participation in randomised cancer treatment trials or interventions aimed at making the process easier or more efficient. A range of study designs were included. However, given the nature of the topic, no relevant indexing terms were available for any of the databases searched, and the search strategy was heavily reliant on textword searching. This meant that the searches were limited to the terms used by authors in the title and abstract fields of each reference. Because of this, there is always the possibility that studies may have been missed.

We focused on interventions to improve participation in trials involving cancer patients. Studies of interventions with other patient groups may provide useful information that might be transferable to cancer treatment trials. Therefore the review may have excluded studies of patients with other conditions that might have highlighted interventions worthy of further investigation with cancer patients.

## Conclusion

A more robust evidence-base for strategies to maximise patient participation in cancer trials is required. Preferably RCTs should be used to evaluate the effectiveness of interventions. However, we recognise that there are many practical barriers to carrying out RCTs of interventions to increase trial participation within the setting of a cancer treatment RCT.

There are a number of issues that need to be considered in future studies. The interventions in this field are effectively complex interventions and would benefit from being treated as such.[30,31] This could include use of qualitative as well as quantitative methods and piloting to define the intervention. Similar methods could be used to assess whether the intervention is being used in the appropriate context in terms of the barriers to patient participation in the trial/s being considered. One of the included studies effectively used such an approach to investigate the barriers to patient participation specific to the cancer trial.[20] Examples of such approaches are available in other areas of research.[32,33]

The risk of contamination between the experimental and comparison intervention needs to be assessed and taken into consideration. Using cluster randomised trials or increasing sample size are possible approaches[34,35] The possibility of clustering where more than one health professional delivers the intervention also needs to be taken into consideration when estimating the required sample size.[36] The problem raised by a lack of blinding of healthcare professionals cannot be avoided as blinding is not possible in these studies. However measures could perhaps be taken to systematically document the implementation of the intervention and comparison.

Future studies also need to consider the potential influence of social and ethnic background and disease related factors such as severity or stage of disease, social and ethnic background on the effectiveness of interventions to increase patient participation in cancer trials. Such information was frequently not provided in the studies included in this review.

Given the paucity of studies investigating interventions targeted specifically at cancer patients, it would be helpful to consider inclusion of interventions with different patient groups in future updates. It may also be beneficial to examine whether interventions to improve recruitment to nonrandomised trials exist which may be applicable to randomised trials.

The majority of included studies examined interventions targeted at the informed consent process. Where this process is the target of an intervention, trial participation cannot be considered in isolation from the quality of the informed consent process. The dangers of coercion when tailoring the information to maximise patient trial participation rates requires careful consideration.[20,37,38] Work has been carried out to develop a questionnaire to assess the quality of the informed consent process.[39] Some of the included studies assessed understanding or knowledge as well as trial participation as an outcome. However the extent to which understanding or knowledge are an indicator for the quality of the consent process is unclear. Future primary studies directed at the informed consent process should consider assessing the quality of the process as well as the impact on participation.

## Competing interests

The author(s) declare that they have no competing interests.

## Authors' contributions

CMcD was responsible for writing the protocol, study selection, data extraction, validity assessment and writing the final report. ZH was involved in all of these processes. AE and DAF provided input at all stages and commented on various drafts of the report. LS devised the search strategy and carried out the literature searches. CMcD is guarantor for the paper.

## Supplementary Material

Additional File 1 Search strategy Click here for file
