# Supplementary material for: Increasing participation of cancer patients in randomised controlled trials: a systematic review
Source: Trials. 2006 May 17;7:16. doi: 10.1186/1745-6215-7-16 (PMC1489947; doi:10.1186/1745-6215-7-16)
Supplement: Additional File 1 — Search strategy [file 1745-6215-7-16-S1.doc]

**Search strategy**

**MEDLINE - Ovid host**

1966 - Wk 3 Nov 2004 (1206 records)

**Searched:** 07/01/05

**MEDLINE In-Process - Ovid host**

Jan 2005 (40 records)

**Searched:** 07/01/05

1. exp NEOPLASMS/
2. (cancer$ or tumor$ or tumour$ or malignan$ or oncolog$ or carcinoma$ or neoplas$).ti,ab.
3. 1 or 2
4. ((increas$ or improv$ or motivat$ or encourag$ or influenc$ or effect$ or affect$ or attract$ or endors$ or promot$ or facilitat$ or enhanc$ or challeng$ or refus$ or reluctan$) adj2 (accru$ or recruit$ or enrol$ or particip$ or enlist$ or join$ or enter or enters or entered or entry) adj2 (trial$ or study or studies or research or rct$ or randomi?ed)).ti,ab.
5. ((difficult$ or problem$ or obstacle$ or barrier$ or deter or deters or deterrent or discourag$ or impediment$ or failure) adj2 (accru$ or recruit$ or enrol$ or particip$ or enlist$ or join$ or enter or enters or entered or entry) adj2 (trial$ or study or studies or research or rct$ or randomi?ed)).ti,ab.
6. ((perception$ or perceiv$ or attitude$ or decision$ or process$ or reason$) adj2 (accru$ or recruit$ or enrol$ or particip$ or enlist$ or join$ or enter or enters or entered or entry) adj2 (trial$ or study or studies or research or rct$ or randomi?ed)).ti,ab.
7. ((willing$ or agree$ or consent$ or permission or assent or permit$ or decide$ or deciding) adj2 (accru$ or recruit$ or enrol$ or particip$ or enlist$ or join$ or enter or enters or entered or entry) adj2 (trial$ or study or studies or research or rct$ or randomi?ed)).ti,ab.
8. ((declin$ or unwilling$ or discourag$) adj2 (accru$ or recruit$ or enrol$ or particip$ or enlist$ or join$ or enter or enters or entered or entry) adj2 (trial$ or study or studies or research or rct$ or randomi?ed)).ti,ab.
9. ((strateg$ or method$ or intervention$ or incentive$) adj2 (accru$ or recruit$ or enrol$ or particip$ or enlist$ or join$ or enter or enters or entered or entry) adj2 (trial$ or study or studies or research or rct$ or randomi?ed)).ti,ab.
10. or/4-9
11. 3 and 10
12. exp *Clinical Trials/
13. clinical trial.pt.
14. 12 or 13
15. *Patient Participation/
16. *Patient Selection/
17. *Informed Consent/
18. *Research Subjects/
19. or/15-18
20. 3 and 14 and 19

21. 11 or 20
